# The Type III Secretion System Effector SptP of Salmonella enterica Serovar Typhi

**DOI:** 10.1128/JB.00647-16

**Published:** 2017-01-30

**Authors:** Rebecca Johnson, Alexander Byrne, Cedric N. Berger, Elizabeth Klemm, Valerie F. Crepin, Gordon Dougan, Gad Frankel

**Affiliations:** a MRC Centre for Molecular Bacteriology and Infection, Department of Life Sciences, Imperial College London, London, United Kingdom; b Wellcome Trust Sanger Institute, Wellcome Trust Genome Campus, Hinxton, Cambridge, United Kingdom; Princeton University

**Keywords:** Salmonella enterica serovar Typhi, SptP, type III secretion system

## Abstract

Strains of the various Salmonella enterica serovars cause gastroenteritis or typhoid fever in humans, with virulence depending on the action of two type III secretion systems (Salmonella pathogenicity island 1 [SPI-1] and SPI-2). SptP is a Salmonella SPI-1 effector, involved in mediating recovery of the host cytoskeleton postinfection. SptP requires a chaperone, SicP, for stability and secretion. SptP has 94% identity between S. enterica serovar Typhimurium and *S*. Typhi; direct comparison of the protein sequences revealed that *S*. Typhi SptP has numerous amino acid changes within its chaperone-binding domain. Subsequent comparison of Δ*sptP S*. Typhi and S. Typhimurium strains demonstrated that, unlike SptP in S. Typhimurium, SptP in *S*. Typhi was not involved in invasion or cytoskeletal recovery postinfection. Investigation of whether the observed amino acid changes within SptP of *S*. Typhi affected its function revealed that *S*. Typhi SptP was unable to complement S. Typhimurium Δ*sptP* due to an absence of secretion. We further demonstrated that while S. Typhimurium SptP is stable intracellularly within *S*. Typhi, *S*. Typhi SptP is unstable, although stability could be recovered following replacement of the chaperone-binding domain with that of S. Typhimurium. Direct assessment of the strength of the interaction between SptP and SicP of both serovars via bacterial two-hybrid analysis demonstrated that *S*. Typhi SptP has a significantly weaker interaction with SicP than the equivalent proteins in S. Typhimurium. Taken together, our results suggest that changes within the chaperone-binding domain of SptP in *S*. Typhi hinder binding to its chaperone, resulting in instability, preventing translocation, and therefore restricting the intracellular activity of this effector.

**IMPORTANCE** Studies investigating Salmonella pathogenesis typically rely on Salmonella Typhimurium, even though Salmonella Typhi causes the more severe disease in humans. As such, an understanding of S. Typhi pathogenesis is lacking. Differences within the type III secretion system effector SptP between typhoidal and nontyphoidal serovars led us to characterize this effector within *S*. Typhi. Our results suggest that SptP is not translocated from typhoidal serovars, even though the loss of *sptP* results in virulence defects in S. Typhimurium. Although SptP is just one effector, our results exemplify that the behavior of these serovars is significantly different and genes identified to be important for S. Typhimurium virulence may not translate to *S*. Typhi.

## INTRODUCTION

Salmonella species are diverse Gram-negative intracellular pathogens responsible for a range of diseases resulting in significant morbidity and mortality among both animals and humans worldwide (1). In humans, the outcome of infection with Salmonella primarily depends on the infecting serovar; while nontyphoidal serovars, such as Salmonella enterica serovar Typhimurium, typically cause self-limiting gastroenteritis, typhoidal serovars, such as Salmonella enterica serovar Typhi and Salmonella enterica serovar Paratyphi, result in the invasive systemic disease typhoid fever (1).

Central to Salmonella virulence is the action of two type III secretion systems (T3SS), encoded on Salmonella pathogenicity island 1 (SPI-1) and SPI-2, which secrete over 40 effectors to subvert host cell processes during infection (2–4). The SPI-1 T3SS is active when Salmonella is extracellular, where it functions to permit the invasion of nonphagocytic host cells (4), while the SPI-2 T3SS is activated upon internalization, where it functions to create a stable and permissive intracellular niche, termed the Salmonella-containing vacuole (SCV) (3, 4).

Although *S*. Typhi causes a more serious disease in humans, *S*. Typhimurium is often used as the model for understanding Salmonella pathogenesis, owing to biosafety concerns and, most importantly, the availability of viable *in vivo* models, since *S*. Typhi and other typhoidal serovars are strictly restricted to humans (5). Despite the widespread acceptance of *S*. Typhimurium as the Salmonella model organism, significant genomic differences exist between *S*. Typhimurium and *S*. Typhi. Comparison of *S*. Typhimurium strain LT2 to *S*. Typhi reference strain CT18 revealed that 89% of genes were shared; approximately 480 genes were unique to LT2 and approximately 600 genes were unique to CT18 (6). Significant variations in prophage, pathogenicity island, and plasmid elements were observed between the two serovars (6).

Importantly, many known SPI-1 and SPI-2 T3SS effectors are absent or pseudogenes in *S*. Typhi, and this may be associated with genome degradation linked with host restriction (6) and could contribute to the differences in pathogenicity observed between typhoidal and nontyphoidal serovars. A notable example is the *S*. Typhimurium effector GtgE, which is absent in *S*. Typhi (6); the introduction of GtgE into *S*. Typhi permits its growth in nonpermissive mouse cells, strongly linking the absence of this effector to typhoidal host restriction (7). Another example which highlights the limitations of using *S*. Typhimurium to investigate Salmonella pathogenesis is the fact that the Salmonella virulence plasmid (pSLT), encoding the effectors SpvB and SpvC, is required for full *S*. Typhimurium virulence in mice (8) but absent in *S*. Typhi and other typhoidal serovars (5, 9). Additional effectors that are either absent or pseudogenes in *S*. Typhi (CT18) include SopA, CigR, SopE2, SlrP, SseJ, SopD2, AvrA, SteB, GogB, SseI/SrfH, SseK1, SseK2, SseK3, and SspH1 (6, 10). Other effectors appear to be differentially evolved between human-restricted and generalist serovars, including SipD, SseC, SseD, SseF, SifA, and SptP (11), which could reflect functional differences. Further evidence of the degradation of the T3SS effector repertoire has been reported within the highly successful and globally dominant *S*. Typhi haplotype 58 (H58), which, owing to a premature stop codon at position 185, lacks both the GTPase-activating phosphatase (GAP) and tyrosine phosphatase domains of SptP and therefore lacks a functional copy of this effector (12).

SptP is an SPI-1 effector encoded within the SPI-1 pathogenicity island immediately downstream of its chaperone, SicP (13). Like other effectors, SptP requires its chaperone for stability within the bacterial cytosol and to direct secretion (13, 14). SptP is a modular protein consisting of three distinct domains: a chaperone-binding domain, a GAP domain, and a tyrosine phosphatase domain. SptP is best characterized in its role as a GTPase-activating protein, acting antagonistically to another SPI-1 effector, SopE/SopE2, a guanine nucleotide exchange factor (GEF) which manipulates the host cytoskeleton to permit membrane ruffle formation and Salmonella internalization (2, 15). SptP deactivates the Rho GTPases Rac1 and Cdc42 to reverse cytoskeletal changes and return the host membrane to a steady-state condition (16). Importantly, extended activation of Rho GTPases by SopE can be sensed by host cells by NOD1, resulting in NF-κB activation and proinflammatory signaling (17); antagonism of SopE by SptP therefore represents an important mechanism to prevent the activation of host immune defenses. SptP has also been reported to directly inhibit the activation of the mitogen-activated protein kinase pathway and subsequently downregulate tumor necrosis factor alpha secretion from infected J774A.1 macrophages (18). The less well characterized tyrosine phosphatase domain of SptP has been shown to be involved in SCV biogenesis and bacterial replication up to 8 h postinfection (19). SptP is also important for virulence *in vivo*, as a Δ*sptP S*. Typhimurium SL1344 strain demonstrates attenuated virulence in BALB/c mice (20) and disruption of *sptP* in *S*. Typhimurium ST4/74 also results in reduced intestinal colonization of chicks, pigs, and cattle (21).

Most of the characterization of SptP has been performed with *S*. Typhimurium (16, 22–24), with the underlying assumption being that *S*. Typhi behaves similarly. The phenotypes attributed to SptP in *S*. Typhimurium, in particular, the virulence defects reported during *in vivo* infection, would seemingly contradict the global success of H58 *S*. Typhi strains, which lack a functional copy of SptP (12). This suggests that the function of this effector in *S*. Typhi may be different from that reported in *S*. Typhimurium.

## RESULTS

### Comparison of SptP from different serovars.

**FIG 1:**
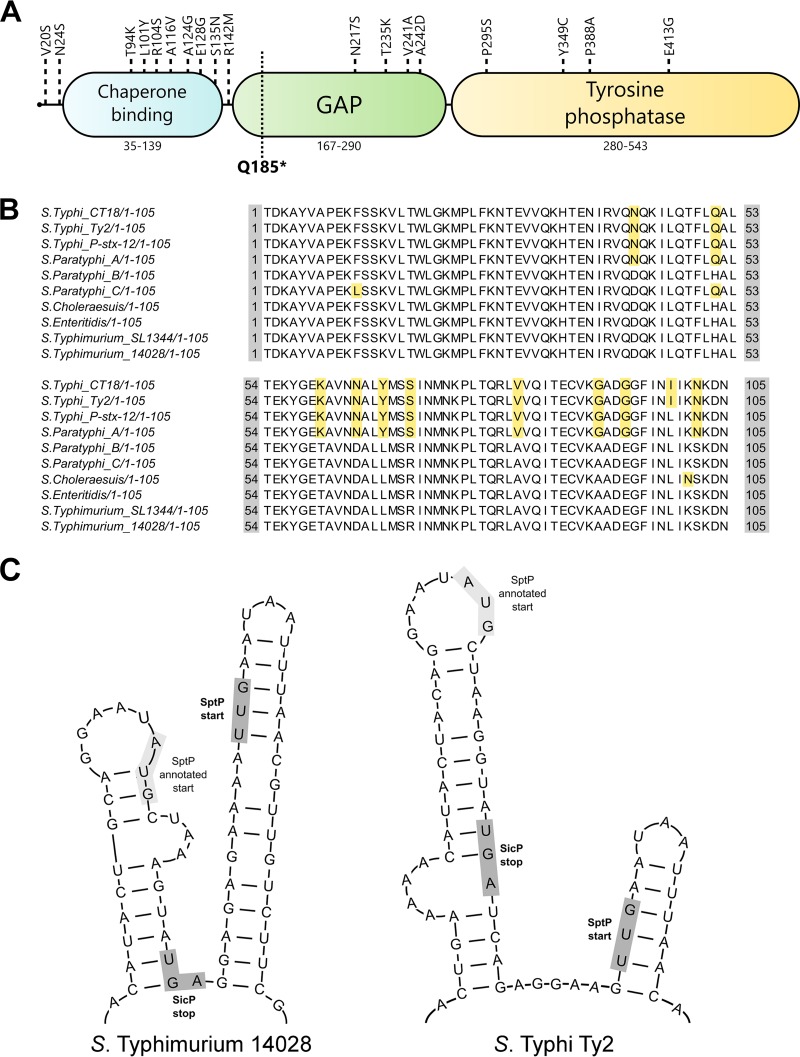
SptP of Salmonella Typhi. (A) Schematic of SptP showing amino acid changes (weakly similar or dissimilar amino acids) between *S*. Typhimurium 14028 and *S.* Typhi Ty2. The location of the single nucleotide polymorphism (Q185*) which generates a premature stop codon in H58 isolates is also shown. (B) Alignment of the sequence of the chaperone-binding domain (positions 35 to 139) of SptP from various Salmonella serovars. Nonconserved amino acids are highlighted. (C) Comparison of the mRNA structures upstream of the start codon of SptP present in *S*. Typhimurium and *S.* Typhi, predicted by use of the Mfold server. Both the annotated ATG (AUG) start codon and the proposed TTG (UUG) start codon of SptP are highlighted.

SptP is relatively conserved between *S*. Typhimurium and *S*. Typhi, sharing 94% amino acid sequence identity between the two serovars. Alignment of the amino acid sequences of SptP from *S*. Typhi Ty2 and SptP from *S*. Typhimurium 14028 revealed that a large number of amino acid changes (10/31) are concentrated within the chaperone-binding domain of SptP (residues 35 to 139 [25]) (Fig. 1A), despite SicP being highly conserved between serovars (97% identity). Direct comparison of the chaperone-binding domains of SptP from various Salmonella serovars shows that these amino acid changes are conserved between *S*. Typhi and *S*. Paratyphi A and that these amino acids are distinct from those in nontyphoidal serovars, including *S*. Typhimurium, *S*. Enteritidis, and *S*. Choleraesuis (Fig. 1B).

Further differences between *sptP* of *S*. Typhimurium and *S*. Typhi can be found outside the protein-coding sequence. It has previously been demonstrated in *S*. Typhimurium that efficient translation of SptP following transcription from its endogenous locus depends on disruption of a stem-loop approximately 20 nucleotides upstream of the start codon of SptP, which is mediated by the upstream translation of SicP (23). Elongation of this stem-loop by just 3 nucleotides decreases the level of translation of SptP by approximately 80% (23). Comparison of the predicted RNA structure (by use of the Mfold server) (26) of this region between the two serovars reveals that *S*. Typhi possesses an elongated stem-loop structure relative to the sequence of *S*. Typhimurium (40 bp and 27 bp, respectively) (Fig. 1C), suggesting that the expression of SptP at the level of translation may differ between the two serovars. Given these differences, we investigated if SptP in *S*. Typhi has the same functions reported during *S*. Typhimurium infection.

### The role of SptP in Salmonella invasion and cytoskeletal recovery.

In *S*. Typhimurium, the loss of *sptP* abolishes the ability of infected cells to recover the host cytoskeleton, with cells displaying extended actin disruption up to 3 h postinfection; conversely, cells infected with wild-type (WT) *S*. Typhimurium regain a normal cytoskeletal structure as soon as 80 min postinfection (16). To assess if the same phenotype was seen during *S*. Typhi infection, we compared the ability of HeLa cells to regain a normal actin cytoskeleton following infection with WT and Δ*sptP S*. Typhi strains. The equivalent *S*. Typhimurium strains were used as controls.

**FIG 2:**
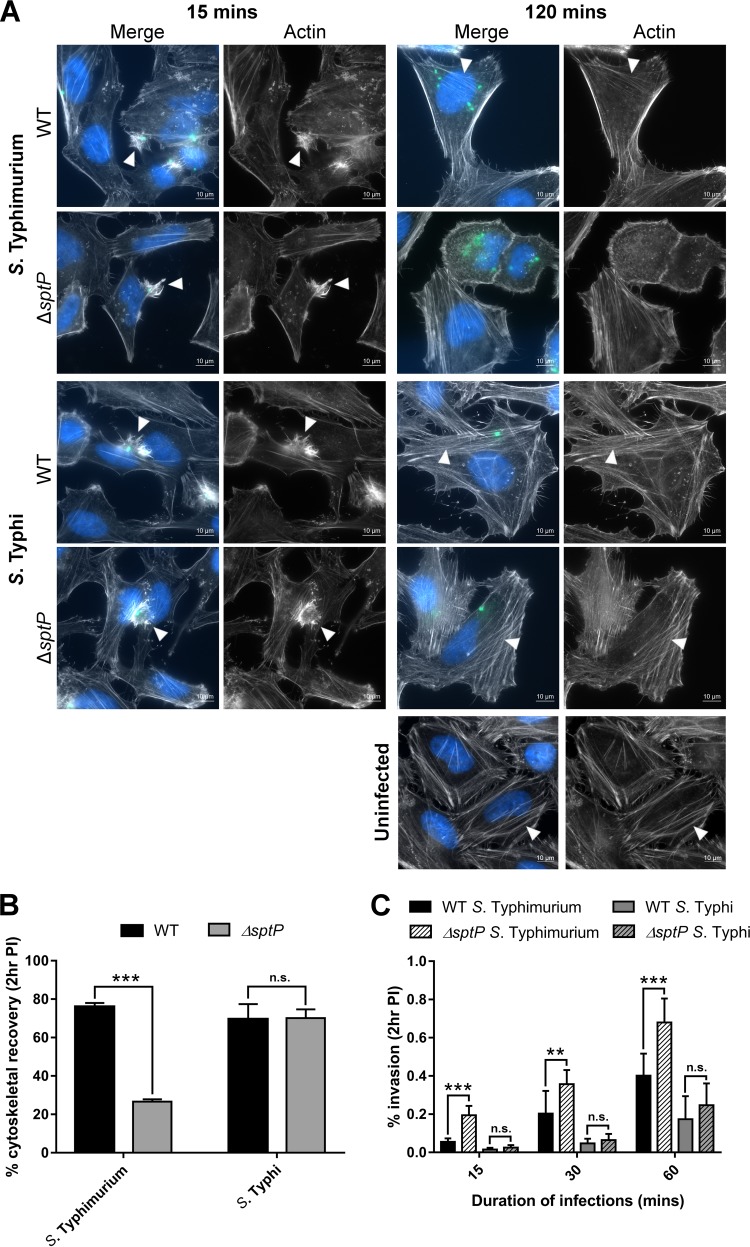
Role of SptP in invasion and cytoskeletal recovery following exponential growth. Salmonella strains, grown aerobically to late exponential phase, were used to infect HeLa cells at an MOI of 100. (A) Representative images of HeLa cells infected with WT or Δ*sptP S*. Typhimurium or *S*. Typhi at 15 min and 2 h postinfection. Actin was stained with phalloidin (white), bacteria were stained with the CSA-1 antibody (green), and nuclei were stained with Hoechst (blue). Arrowheads, invasion-associated membrane ruffles (15 min) or stress fibers (120 min). (B) HeLa cells infected with Salmonella strains were fixed at 2 h postinfection (PI) and stained with phalloidin to determine the proportion of infected cells displaying a normal actin cytoskeleton. At least 100 infected cells were counted per strain and per biological repeat. Error bars show SDs (*n* = 3). The levels of cytoskeletal recovery of WT and Δ*sptP* strains were compared by *t* test (***, *P* < 0.001; n.s., no significant difference). (C) HeLa cells were infected with WT or Δ*sptP S*. Typhimurium 14028 or *S*. Typhi Ty2 for 15, 30, or 60 min. The percentage of intracellular bacteria at 2 h postinfection relative to the number of bacteria added in the inoculum is shown. Error bars show SDs (*n* = 3). The invasion rates of the strains were compared by *t* test (**, *P* < 0.01; ***, *P* < 0.001).

During epithelial cell infection, both *S*. Typhimurium and S. Typhi manipulate the host cytoskeleton, with membrane ruffles associated with invasion events clearly being visible at 15 min postinfection (Fig. 2A). By 2 h, cells infected with WT Salmonella display an actin architecture similar to that seen for uninfected cells, visible as the formation of stress fibers (Fig. 2A). In line with previous findings (16), cells infected with *S*. Typhimurium Δ*sptP* did not demonstrate cytoskeletal recovery and continued to exhibit membrane ruffling and an absence of stress fibers. In contrast, cells infected with *S*. Typhi Δ*sptP* displayed a recovered cytoskeleton at 2 h postinfection. To quantify this phenotype, we determined the proportion of infected cells displaying an actin architecture akin to that of uninfected cells at 2 h postinfection. While 76% of cells infected with WT *S*. Typhimurium (*n* = 309) demonstrated cytoskeletal recovery, only 27% of cells infected with *S*. Typhimurium Δ*sptP* (*n* = 315) demonstrated a recovered actin cytoskeleton. In comparison, 70% of cells infected with either WT *S*. Typhi (*n* = 294) or *S*. Typhi Δ*sptP* (*n* = 301) exhibited cytoskeletal recovery (Fig. 2B).

Although SptP has not previously been linked with invasion in *S*. Typhimurium (20, 27), SptP, via its antagonism of SopE, should directly govern the size and duration of membrane ruffling and therefore influence the efficiency of invasion. As such, we compared the levels of invasion of WT and Δ*sptP S*. Typhimurium and *S*. Typhi strains into HeLa cells. While deletion of *sptP* from *S*. Typhimurium resulted in a significantly increased level of invasion relative to that of the WT strain, deletion of *sptP* from *S*. Typhi did not affect the invasion efficiency (Fig. 2C). Increasing the length of time that Salmonella was incubated with host cells improved the invasion efficiency of all strains but did not alter the trends observed (Fig. 2C). SPI-1-deficient *S*. Typhimurium (Δ*prgH*) and *S*. Typhi (Δ*invA*) strains, used as controls, failed to invade HeLa cells, as expected (see Fig. S1 in the supplemental material).

### Growth under different SPI-1-inducing conditions influences SopE activity in *S*. Typhi.

A recent study comparing *S*. Paratyphi A and *S*. Typhimurium following growth under different SPI-1-inducing conditions demonstrated that *S*. Paratyphi was significantly less invasive, had reduced levels of SPI-1 expression, and had reduced levels of SptP and SopE2 following aerobic growth to late exponential phase compared to the results obtained by microaerobic growth to stationary phase (28). Since our initial comparisons between WT and Δ*sptP* Salmonella strains were performed following aerobic exponential growth (Fig. 2), we were interested in whether SopE, the guanine exchange factor which SptP antagonizes (16), was active during *S*. Typhi infection after growth under these conditions.

**FIG 3:**
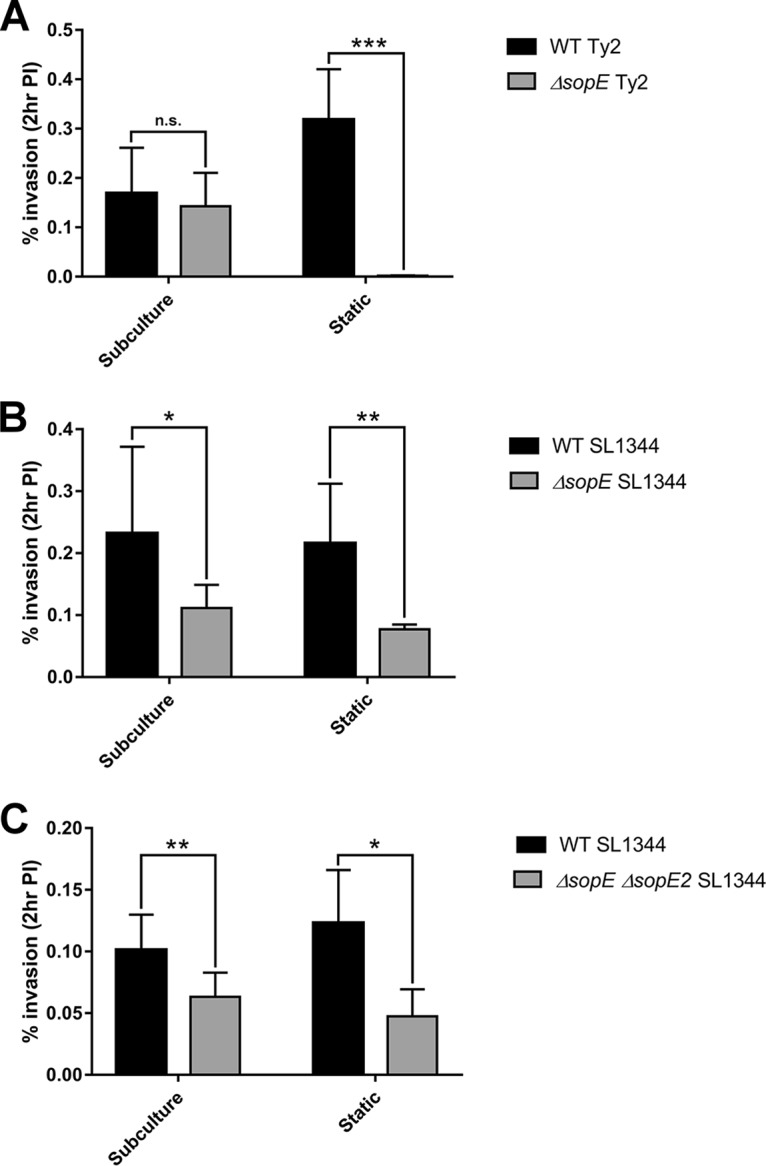
Growth conditions alter the dependency on SopE for invasion in *S*. Typhi. Salmonella strains were grown either aerobically to late exponential phase (subculture) or to stationary phase under microaerobic conditions (static overnight culture) and added to HeLa cells at an MOI of 100. Cells were infected for 1 h with WT and Δ*sopE S*. Typhi (A) and 15 min for WT, Δ*sopE*, and Δ*sopE* Δ*sopE2 S*. Typhimurium SL1344 (B and C). The percentage of intracellular bacteria at 2 h postinfection relative to the number of bacteria added in the inoculum is shown. Error bars show SDs (*n* = 3). The invasion rates of the strains were compared by *t* test (*, *P* < 0.05; **, *P* < 0.01; ***, *P* < 0.001; n.s., no significant difference).

To determine if environmental conditions impact SopE activity, we assessed the invasiveness of WT or Δ*sopE S*. Typhi and *S*. Typhimurium strains following aerobic growth to late exponential phase or microaerobic growth to stationary phase. Since *S*. Typhimurium 14028 expresses SopE2 but lacks SopE (29), while *S*. Typhi Ty2 expresses SopE but not SopE2 (6, 30), we instead used the *S*. Typhimurium strain SL1344, which has both SopE and SopE2 (31), for the comparisons in order to account for any differences in activity or regulation between SopE and SopE2 (31). Following growth to late exponential phase, deletion of *sopE* in *S*. Typhi resulted in a modest decrease in the rate of invasion into HeLa cells, with a relative invasion rate of 84.4% of that of the WT (Fig. 3A). When grown to stationary phase, however, invasion of the *S*. Typhi Δ*sopE* strain was significantly attenuated (0.002% invasion efficiency, with a relative invasion rate of 0.5% of that of the WT) (Fig. 3A). A Δ*invA S*. Typhi strain was unable to invade HeLa cells following growth to either exponential phase or stationary phase, demonstrating that invasion under both conditions is SPI-1 dependent (Fig. S1). In contrast, *S*. Typhimurium SL1344 did not demonstrate this marked phenotypic difference between growth conditions, as the Δ*sopE* (Fig. 3B) and Δ*sopE* Δ*sopE2* (Fig. 3C) strains demonstrated similar reductions in the rates of invasion following growth to either late exponential or stationary phase.

**FIG 4:**
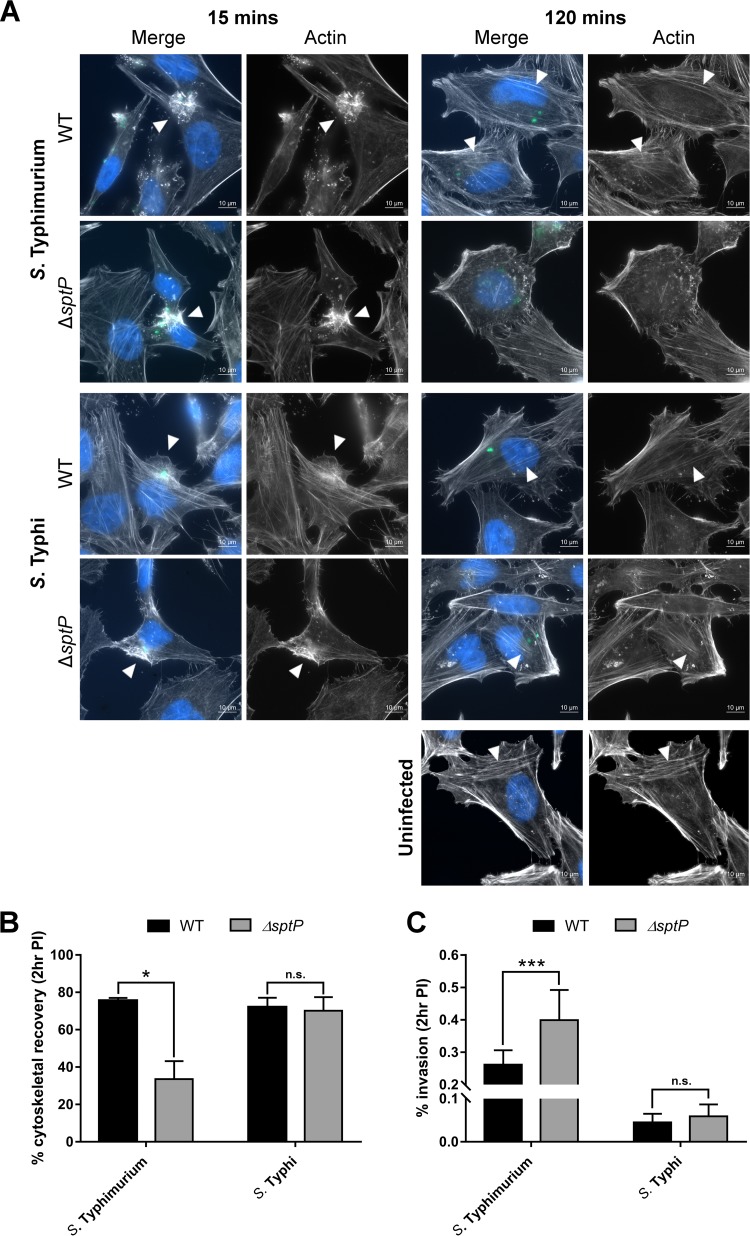
Role of SptP in invasion and cytoskeletal recovery following growth to stationary phase. Salmonella strains, grown under microaerobic conditions to stationary phase, were used to infect HeLa cells at an MOI of 100. (A) Representative images of HeLa cells infected with WT *or* Δ*sptP S*. Typhimurium 14028 or *S*. Typhi Ty2 at 15 min and 2 h postinfection. Actin was stained with phalloidin (white), bacteria were stained with the CSA-1 antibody (green), and nuclei were stained with Hoechst (blue). Arrowheads, invasion-associated membrane ruffles (15 min) or stress fibers (120 min). (B) HeLa cells infected with Salmonella strains were fixed at 2 h postinfection and stained with phalloidin to determine the proportion of infected cells displaying a normal actin cytoskeleton. At least 100 infected cells were counted per strain and per biological repeat. Error bars show SDs (*n* = 3). The levels of cytoskeletal recovery of WT and Δ*sptP* strains were compared by *t* test (*, *P* < 0.05; n.s., no significant difference). (C) HeLa cells were infected with WT *or* Δ*sptP S*. Typhimurium 14028 or *S*. Typhi Ty2 for 30 min. The percentage of intracellular bacteria at 2 h postinfection relative to the number of bacteria added in the inoculum is shown. Error bars show SDs (*n* = 3). The invasion rates of the strains were compared by *t* test (***, *P* < 0.001).

Since these results suggest that SopE is nonessential for *S*. Typhi invasion following aerobic subculture to late exponential phase, it follows that SptP would also be dispensable, potentially explaining the lack of phenotypes previously observed for *S*. Typhi Δ*sptP* compared to *S*. Typhimurium Δ*sptP* (Fig. 2). The role of SptP in invasion and cytoskeletal recovery postinfection was therefore reassessed as before, but with strains instead being grown under microaerobic conditions to stationary phase. While significant differences in invasion and cytoskeletal recovery were observed between the WT and Δ*sptP S*. Typhimurium strains, no significant differences between the WT and Δ*sptP S*. Typhi strains were observed (Fig. 4).

### Complementation of *S*. Typhimurium Δ*sptP* with S. Typhi *sptP*.

**FIG 5:**
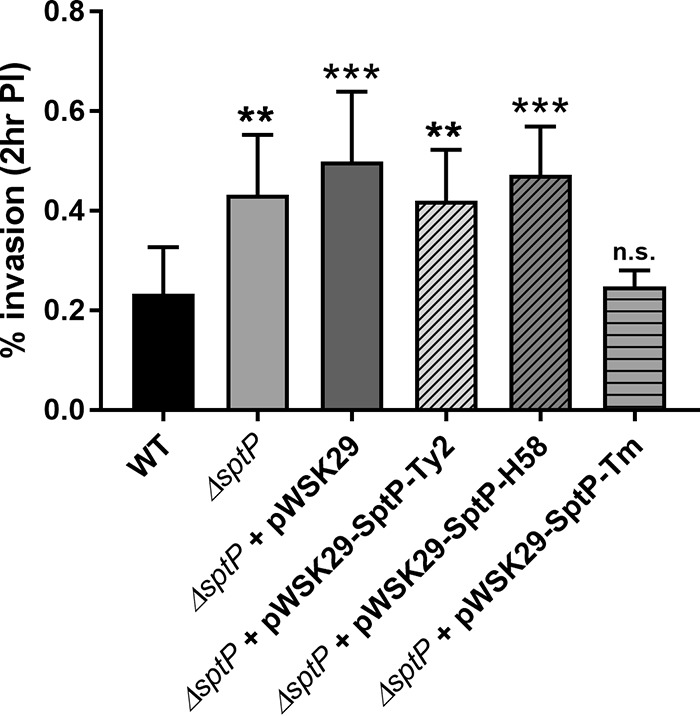
Expression of *S*. Typhi SptP fails to complement the deletion in *S*. Typhimurium. WT and Δ*sptP S*. Typhimurium strains complemented with either empty pWSK29-Spec or plasmids expressing *S*. Typhimurium (Tm) or *S*. Typhi Ty2 SptP from a constitutive promoter were grown aerobically to late exponential phase and added to HeLa cells at an MOI of 100. Strains were left to infect the cells for 15 min, and the number of intracellular bacteria was determined at 2 h postinfection. The percentage of intracellular bacteria relative to the number of bacteria added in the inoculum is shown. Error bars show SDs (*n* = 3). The invasion rates of the strains were compared to the rate for the WT by one-way ANOVA, followed by Tukey's *post hoc* test (**, *P* < 0.01; ***, *P* < 0.001; n.s., no significant difference).

Since the loss of SptP does not result in phenotypes during *S*. Typhi infection the same as those observed during *S*. Typhimurium infection, it suggests that functional differences exist between the SptP proteins from these serovars. SptP was one of several Salmonella T3SS proteins identified as being differentially evolved between typhoidal and nontyphoidal serovars (11). We therefore investigated if expression of *S*. Typhi SptP would be able to cross complement *S*. Typhimurium Δ*sptP*. As shown above (Fig. 2 and 4), deletion of *sptP* from *S*. Typhimurium results in a significant increase in the level of invasion of HeLa cells. While complementation with *S*. Typhimurium *sptP* restored the level of invasion back to WT levels, *S*. Typhimurium Δ*sptP* expressing *sptP* from *S*. Typhi was as invasive as both the Δ*sptP* strain and the Δ*sptP* strain carrying the empty pWSK29 vector (Fig. 5). Furthermore, a Δ*sptP S*. Typhimurium strain expressing the truncated SptP present within H58 strains was as invasive as the strain expressing Ty2 SptP (Fig. 5).

**FIG 6:**
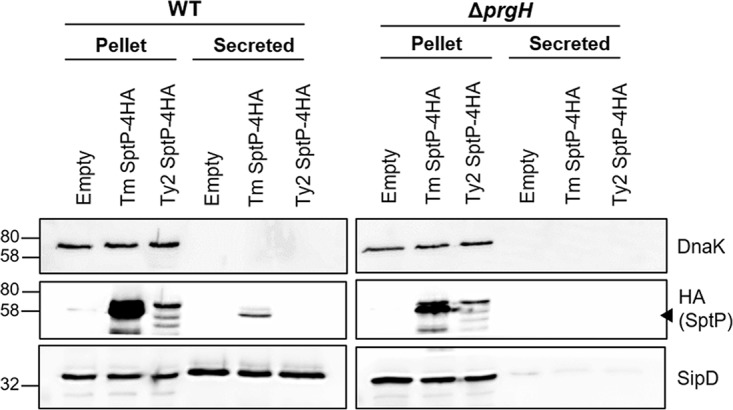
*S*. Typhimurium cannot secrete S. Typhi SptP. WT and SPI-1 mutant *S*. Typhimurium (Δ*prgH*) strains expressing C-terminally 4HA-tagged SptP were grown to late exponential phase. The presence of S. Typhi Ty2 or *S*. Typhimurium (Tm) SptP in bacterial pellets and the supernatant was detected using an anti-HA antibody; the cytoplasmic protein DnaK was used to control for bacterial lysis, and the SPI-1 translocon component SipD was used as a positive control for SPI-1 secretion. A representative blot of three independent repeats is shown. The numbers on the left are molecular masses (in kilodaltons). Arrowhead, the band which corresponds to SptP.

The inability of *S*. Typhi SptP to complement *S*. Typhimurium Δ*sptP* could be due to either the amino acid changes within the GAP and tyrosine phosphatase domains rendering it functionally inactive or the numerous substitutions within the chaperone-binding region compromising the ability of *S*. Typhi SptP to bind the SicP chaperone, therefore destabilizing SptP intracellularly and preventing its secretion by the SPI-1 T3SS (13, 14) (Fig. 1). We tested these two possibilities by performing an SPI-1 secretion assay with hemagglutinin (HA)-tagged SptP expressed in *S*. Typhimurium. While SptP from both *S*. Typhimurium and *S*. Typhi was detected intracellularly in WT *S*. Typhimurium, *S*. Typhi SptP was observed at lower levels and only *S*. Typhimurium SptP was detected in culture supernatants, with secretion being dependent on the presence of a functional SPI-1 T3SS (Fig. 6). Similar levels of the SPI-1 translocon component SipD were secreted by the WT strains, suggesting that the lack of *S*. Typhi SptP secretion relates specifically to differences between the two SptP variants, likely from changes within the chaperone-binding domain.

### The chaperone-binding domain of SptP is essential for stability.

Although SicP is highly similar between *S*. Typhimurium and *S*. Typhi (97% identity), the inability of *S*. Typhi SptP to function within *S*. Typhimurium might reflect its inability to efficiently bind *S*. Typhimurium SicP, rather than indicating a general inability to bind its chaperone. As SicP binding of SptP also prevents the degradation of SptP within the bacterial cytosol prior to secretion (13), the ability of SptP to bind SicP can be assessed by detecting the total levels of HA-tagged *S*. Typhi and *S*. Typhimurium SptP in an *S*. Typhi SPI-1 mutant background (Δ*invA*), where secretion of effectors is prevented. Two constructs were used to assess SptP stability for both *S*. Typhimurium and *S*. Typhi SptP: one constitutively expressing SptP from the T3 promoter of pWSK29 and one expressing SptP from the endogenous promoter of each serovar, taken to be 700 bp upstream from the SptP start codon, on the basis of the predicted transcriptional start site from *S*. Typhimurium transcriptome sequencing data (32). While the T3 promoter accounts for any regulatory differences between *S*. Typhi and *S*. Typhimurium, expression from the endogenous promoter, which also encodes SicP, ensures the correct coregulation of SicP and SptP at both the transcriptional and translational levels (23).

**FIG 7:**
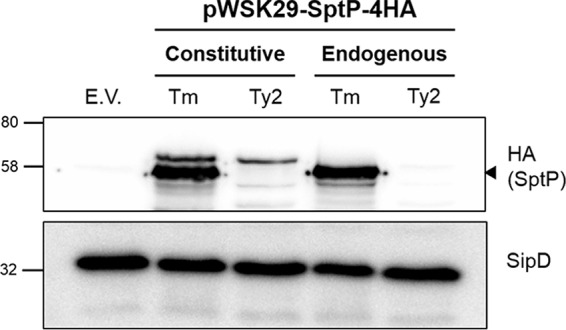
SptP is unstable within *S*. Typhi. SPI-1 mutant (Δ*invA*) *S*. Typhi bacteria expressing *S*. Typhimurium (Tm) or *S*. Typhi Ty2 SptP-4HA from either a constitutive T3 promoter or the endogenous promoter of *sptP* were grown aerobically to late exponential phase. The presence of intracellular SptP was detected using an anti-HA antibody. SipD was used as both a loading control and a positive control for SPI-1 expression. A representative blot of three independent repeats is shown. The numbers on the left are molecular masses (in kilodaltons). Arrowhead, the band which corresponds to SptP; E.V., empty vector.

*S*. Typhi SptP-4HA was not detectable within bacteria following expression from either the constitutive or endogenous promoter, while SptP-4HA from *S*. Typhimurium was readily detectable following expression from either construct (Fig. 7). The stability of *S*. Typhimurium SptP-4HA within *S*. Typhi indicates that SicP is still functional in this serovar, suggesting that the instability of *S*. Typhi SptP results from changes within SptP alone. It is worth highlighting that the doublet produced from the constitutive SptP-4HA construct likely arises from forced expression from the current annotated ATG start codon of SptP, as well as expression from a downstream TTG codon, reported to be the true start site for SptP (23). Given that only the lower band was secreted (Fig. 6) and the size of the *S*. Typhimurium product expressed by the endogenous promoter also corresponds to that of the lower band, it is probable that the smaller band represents the correct form of SptP.

**FIG 8:**
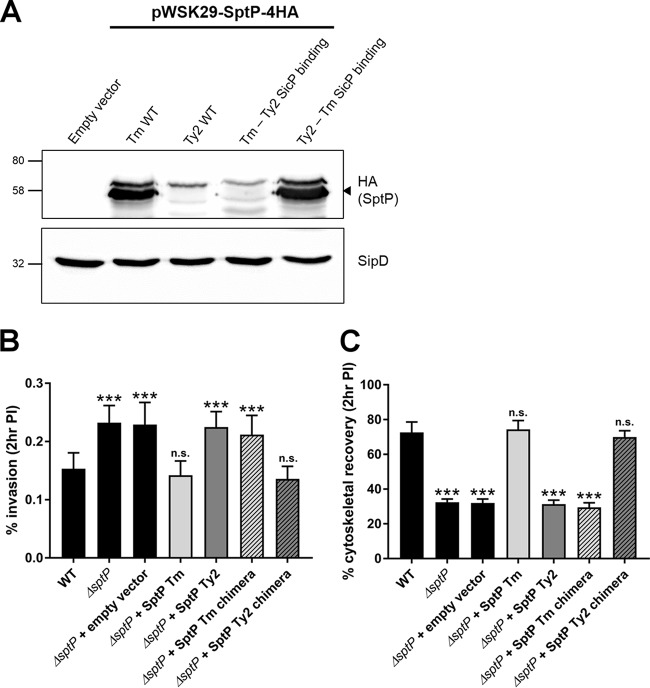
The chaperone-binding domain of *S*. Typhi SptP is responsible for its instability. (A) SPI-1 mutant (Δ*invA*) *S*. Typhi bacteria constitutively expressing either WT SptP-4HA from *S*. Typhimurium (Tm) or *S*. Typhi Ty2 or SptP-4HA chimeras with the SicP binding domain switched with that of the other serovar were grown aerobically to late exponential phase. The presence of SptP was determined using an antibody against the HA tag. SipD was used as both a loading control and a positive control for SPI-1 expression. A representative blot of three independent repeats is shown. The numbers on the left are molecular masses (in kilodaltons). Arrowhead, the band which corresponds to SptP. (B and C) HeLa cells were infected with WT or Δ*sptP S*. Typhimurium strains constitutively expressing either WT SptP-4HA from *S*. Typhimurium (Tm) and *S*. Typhi (Ty2) or the SptP-4HA chimeras for 15 min. The percentage of intracellular bacteria at 2 h postinfection relative to the number of bacteria added in the inoculum is shown (B), and the proportion of infected cells displaying normal actin cytoskeleton was enumerated (C). At least 100 infected cells were counted per strain and per biological repeat. Error bars show SDs (*n* = 3). The invasion rates of the strains and cytoskeletal recovery were compared to those for the WT by one-way ANOVA, followed by Tukey's *post hoc* test (***, *P* < 0.001; n.s., no significant difference).

In order to further confirm that the instability of SptP in *S*. Typhi results from changes within the chaperone-binding domain, constitutive HA-tagged SptP chimeras were constructed in which the first 139 residues of *S*. Typhimurium SptP were swapped with those of *S*. Typhi and vice versa, and the proteins were again expressed in an *S*. Typhi SPI-1 null background (Δ*invA*). As before, WT *S*. Typhi SptP was undetectable within bacteria; however, when the SicP binding domain of *S*. Typhi SptP was replaced with that of *S*. Typhimurium, *S*. Typhi SptP could be detected intracellularly (Fig. 8A). In contrast, *S*. Typhimurium SptP was destabilized when given the *S*. Typhi SicP binding domain, while the WT SptP remained readily detectable (Fig. 8A).

Since replacement of the chaperone-binding domain of SptP confers stability to *S*. Typhi SptP, we were interested to see if these changes would also influence the activity of *S*. Typhi SptP. Therefore, Δ*sptP S*. Typhimurium was transformed with both WT and chimera *S*. Typhimurium and *S*. Typhi HA-tagged SptP, and invasion and cytoskeletal recovery assays were performed. While expression of *S*. Typhimurium SptP-4HA decreased the level of invasion back to WT levels, a strain expressing *S*. Typhi SptP-4HA was found to be as invasive as both the Δ*sptP* strain and the Δ*sptP* strain carrying the empty pWSK29 vector (Fig. 8B), in line with our previous findings (Fig. 5). The same pattern was observed with cytoskeletal recovery, with the *S*. Typhimurium SptP complemented strain achieving recovery rates akin to those achieved with WT infection, while the strain expressing *S*. Typhi SptP demonstrated significantly impaired cytoskeletal recovery at 2 h postinfection (Fig. 8C). However, Δ*sptP S*. Typhimurium expressing the *S*. Typhimurium SptP chimera displayed significantly increased invasion and significantly reduced cytoskeletal recovery compared to WT *S*. Typhimurium (Fig. 8), demonstrating that the *S*. Typhi chaperone-binding domain abolishes the ability of *S*. Typhimurium SptP to complement *sptP* deletion. Conversely, the Δ*sptP S*. Typhimurium strain was fully complemented by expression of the *S*. Typhi SptP chimera, indicating that the GAP domain of *S*. Typhi SptP is still functional.

### Binding of SptP to SicP.

**FIG 9:**
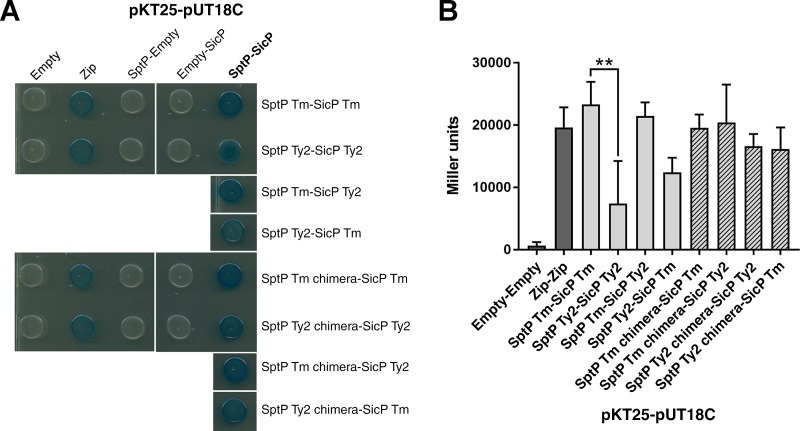
*S*. Typhi SptP interacts weakly with its chaperone, SicP. The interaction of SptP with its chaperone was assessed via a bacterial two-hybrid assay. The adenylate cyclase T25 and T18 fragments were fused to the N terminus of SptP and SicP of *S*. Typhimurium (Tm) and *S*. Typhi Ty2, respectively, and cotransformed into a *cyaA* mutant, E. coli DHM1. The levels of coexpression of the T25 and T18 fragments alone (Empty) or fused to leucine zipper motifs (Zip) were used as negative and positive controls, respectively. (A) Ten microliters of an overnight culture was spotted in triplicate on LB–X-Gal plates and incubated at 30°C for 24 h. Blue colonies indicate restored adenylate cyclase activity. A representative plate is shown. (B) Colonies were resuspended, and a β-galactosidase assay was performed in order to quantify the strength of the interaction between SptP and its chaperone, SicP (Miller units). Error bars show SDs (*n* = 3). The levels of β-galactosidase expression induced by *S*. Typhimurium SptP and *S*. Typhi SptP were compared by *t* test (**, *P* < 0.01).

Assessment of the secretion and stability of SptP within *S*. Typhimurium and *S*. Typhi represents only an indirect way to measure SptP-SicP binding. In order to directly determine the strength of the interaction between SptP and SicP of both serovars, a bacterial two-hybrid assay was performed. SptP from *S*. Typhimurium and *S*. Typhi was found to interact with SicP of both serovars, indicated by the blue coloration of the colonies on Lennox broth (LB)–5-bromo-4-chloro-3-indolyl-β-d-galactopyranoside (X-Gal) indicator plates arising from the reconstitution of adenylate cyclase activity and subsequent induction of β-galactosidase expression (33). However, SptP-SicP interactions involving *S*. Typhi SptP resulted in a color change weaker than that in the equivalent cotransformations involving *S*. Typhimurium SptP (Fig. 9A). Since β-galactosidase expression is correlated with the strength of the interaction, a β-galactosidase assay was performed on the resuspended colonies in order to quantify the ability of SptP and SicP to interact between serovars. While *S*. Typhimurium SptP could interact strongly with SicP from *S*. Typhimurium, the equivalent interaction involving SptP and SicP from *S*. Typhi was significantly weaker (Fig. 9B). Interestingly, the strength of the interaction between *S*. Typhimurium SptP and *S*. Typhi SicP was equivalent to that of the interaction between *S*. Typhimurium SptP and its endogenous chaperone (Fig. 9B), further validating that SicP in *S*. Typhi is still functional.

Although replacement of the *S*. Typhimurium chaperone-binding domain with that of *S*. Typhi does not significantly decrease its interaction with SicP compared to that of WT SptP, replacement of the chaperone-binding domain of *S*. Typhi SptP with that of *S*. Typhimurium increased the ability of *S*. Typhi SptP to bind both *S*. Typhi and *S*. Typhimurium SicP (Fig. 9B), reiterating that the reduced chaperone binding observed within *S*. Typhi SptP likely arises through changes within its SicP binding domain.

## DISCUSSION

The presence of a premature stop codon within the SPI-1 effector SptP in H58 strains (12) led us to characterize the function of this effector during *S*. Typhi infection and compare it to that of this effector during *S*. Typhimurium infection. To date, there has been very limited characterization of SptP or, indeed, any Salmonella T3SS effector using WT *S*. Typhi, with studies instead tending to use *S*. Typhimurium or attenuated *S*. Typhi strains.

Our data demonstrate that SptP of *S*. Typhi has reduced chaperone binding, resulting in intracellular instability and preventing secretion. Unsurprisingly, given the instability of SptP within the Salmonella cytosol, deletion of *sptP* from *S*. Typhi Ty2 did not affect the efficiency of invasion into HeLa cells, in line with the findings of a previous study (24), and did not affect cytoskeletal recovery postinfection. Conversely, *S*. Typhimurium SptP demonstrates strong binding to its chaperone, SicP, ensuring its stability and secretion; as such, deletion of *sptP* from *S*. Typhimurium resulted in significant phenotypic differences, including increased invasion and a failure to recover the host cytoskeleton postinfection, as previously reported (13, 16).

Although significant differences between the ability of *S*. Typhimurium and *S*. Typhi SptP to bind SicP were observed in our bacterial two-hybrid assay, this technique failed to discern any significant differences between the WT and chaperone-binding chimeras of SptP, even though marked phenotypes with the SptP chimera constructs were seen in Salmonella (Fig. 8). The use of Escherichia coli as a host, incubation at a lower temperature (30°C), and overexpression of both SptP and SicP likely promoted protein-protein interactions and reduced the overall sensitivity of the assay. However, experiments conducted in both *S*. Typhimurium and *S*. Typhi demonstrate that changes within the first 139 amino acids of *S*. Typhi SptP, corresponding to the SicP binding domain, are sufficient to compromise its activity.

Our results strongly suggest that SptP is nonfunctional in *S*. Typhi. Interestingly, *S*. Paratyphi A shares the same amino acid changes within the chaperone-binding domain, and SptP failed to be detected intracellularly by Western blotting from *S*. Paratyphi A grown to late exponential phase (28), suggesting that the instability of SptP may be a common feature of typhoidal serovars. This apparent loss of function may explain how a premature stop codon within SptP within strains belonging to the globally dominant *S*. Typhi H58 haplotype is tolerated (12), despite evidence from *in vivo S*. Typhimurium infections demonstrating attenuated virulence when *sptP* is deleted (20, 21). This suggests that *S*. Typhimurium is not always an appropriate model for typhoidal disease and that SptP, like the *spv* locus (8), may be required for systemic disease in mice or other hosts but not humans.

Although pseudogenization or the loss of known SPI-1 and SPI-2 effectors within *S*. Typhi has previously been reported (6, 10), the mechanism by which the function of SptP has been lost—via multiple amino acid changes, reducing its interaction with its chaperone—is unusual. A premature stop codon, such as that found in *S*. Typhi H58 strains (12), or even deletion of the gene would achieve the same end, without expending the energy invested in the transcription or translation of SptP. This could suggest that the loss of SptP function occurred gradually, maybe in conjunction with the acquisition and assimilation of a new effector with a similar function that was better suited to the induction of typhoidal disease; the presence of *S*. Typhi-specific effectors is not unlikely, given that *S*. Typhi CT18 has almost 600 genes not found in *S*. Typhimurium LT2 and effectors found only in *S*. Typhimurium and not *S*. Typhi are common (6). Alternatively, SptP is relatively unique among SPI-1 effectors, in that it is encoded within the SPI-1 pathogenicity island itself (4). Its central location within SPI-1 may not permit deletion or truncation without having deleterious effects on the transcription or translation of neighboring genes, such as *hilA*, thereby driving the loss of function at the protein level.

If SptP is indeed not functional in *S*. Typhi, several key questions remain. Given that extended Rho GTPase activation mediated by SopE during infection would lead to proinflammatory signaling uncharacteristic of *S*. Typhi infection (17, 34), is another effector responsible for antagonizing SopE in *S*. Typhi? Perhaps most intriguingly, the question as to what function SicP, the chaperone of SptP, is performing in *S*. Typhi remains. SicP is relatively conserved between serovars (97% identity); since *S*. Typhimurium SptP is stable within *S*. Typhi and is able to strongly interact with *S*. Typhi SicP in a direct bacterial two-hybrid assay, we can be confident that SicP itself is still functional in *S*. Typhi. It is currently thought that SicP acts as a chaperone for SptP only (35); however, if it is not required to bind SptP in *S*. Typhi, it is unclear why its activity has been preserved. Although SicP is better conserved between serovars than SptP, there are amino acid changes. It is possible that, given time, these changes will accumulate and decrease the activity of SicP. An alternative explanation could be that SicP is simply acting as a chaperone for another, currently unknown effector.

Although the finding is not directly related to the function of SptP, we have also observed that different environmental conditions induced *S*. Typhi-specific regulation of the SPI-1 effector SopE. At present there are two accepted methods to induce SPI-1 expression, achieved either via aerobic growth to late exponential phase (subculture) or through growth to stationary phase under microaerobic conditions (static overnight culture) (28, 36). Our findings suggest that in comparison to *S*. Typhimurium, *S*. Typhi has additional regulatory mechanisms to control SPI-1 activity, likely in response to oxygen tension, acting at least at the level of SopE. Interestingly, a study comparing global SPI-1 expression in *S*. Paratyphi A between the different SPI-1-inducing conditions reported significant differences in SPI-1 expression between aerobic and microaerobic growth (28), while a similar study using *S*. Typhimurium SL1344 did not (36). Overall, this suggests that typhoidal serovars additionally use oxygen availability to tightly control virulence gene expression, an observation that may help explain the different diseases induced by typhoidal and nontyphoidal serovars.

Overall, this study demonstrates that the behavior of *S*. Typhi is significantly different from that of *S*. Typhimurium; the potential loss of function of SptP within *S*. Typhi exemplifies just how different these serovars are, even at the level of a single effector. This indicates that the findings of work performed in one serovar may not directly translate to the other, highlighting the need for further studies to elucidate the role of T3SS effectors in the context of *S*. Typhi infection.

## MATERIALS AND METHODS

### Bacterial strains, growth conditions, and cell culture.

The strains and plasmids used in this study are listed in Table 1. Salmonella strains were routinely grown in Lennox broth (LB; Sigma-Aldrich). Kanamycin (50 μg/ml), ampicillin (100 μg/ml), and spectinomycin (100 μg/ml) were supplemented as required.

**TABLE 1:** Strains and plasmids used in this study

Strain or plasmid	Identifier	Genotype or comments	Source or reference
Strains			
*S*. Typhimurium			
14028	ICC797	WT	This study
14028	ICC1373	Δ*sptP*::Kan^r^	This study
14028	ICC796	Δ*prgH*::Tn*5*	This study
SL1344	ICC314	WT	This study
SL1344	ICC1374	Δ*sopE*::Kan^r^	This study
SL1344	ICC1376	Δ*sopE*::FRT Δ*sopE2*::Kan^r^	This study
*S*. Typhi			
Ty2	ICC1500	WT	
Ty2	ICC1522	Δ*sptP*::Kan^r^	This study
Ty2	ICC1556	Δ*invA*::Kan^r^	This study
Ty2	ICC1555	Δ*sopE*::Kan^r^	This study
Plasmids			
pWSK29-Amp			37
pWSK29-4HA			This study
pWSK29-Spec	pICC2489	Empty vector	This study
pWSK29-Spec	pICC2491	*S*. Typhimurium SptP, T3 promoter	This study
pWSK29-Spec	pICC2490	Ty2 SptP, T3 promoter	This study
pWSK29-Spec	pICC2535	H58 SptP, T3 promoter	This study
pWSK29-Spec-4HA	pICC2493	*S*. Typhimurium SptP-4HA, T3 promoter	This study
pWSK29-Spec-4HA	pICC2492	Ty2 SptP-4HA, T3 promoter	This study
pWSK29-Spec-4HA	pICC2494	*S*. Typhimurium SptP-4HA, endogenous promoter	This study
pWSK29-Spec-4HA	pICC2495	Ty2 SptP-4HA, endogenous promoter	This study
pWSK29-Spec-4HA	pICC2520	*S*. Typhimurium SptP-Ty2 SicP binding 4HA, T3 promoter	This study
pWSK29-Spec-4HA	pICC2521	Ty2 SptP-*S*. Typhimurium SicP binding 4HA, T3 promoter	This study
pKT25	pICC2531	Empty vector	Euromedex
pKT25	pICC2533	Zip	Euromedex
pKT25	pICC2498	*S*. Typhimurium SptP	This study
pKT25	pICC2499	Ty2 SptP	This study
pUT18C	pICC2532	Empty vector	Euromedex
pUT18C	pICC2534	Zip	Euromedex
pUT18C	pICC2504	*S*. Typhimurium SicP	This study
pUT18C	pICC2505	Ty2 SicP	This study
pKD4	pICC893	Kanamycin resistance cassette template plasmid	38
pKD46	pICC1298	Bacteriophage lambda red recombinase plasmid	38
pCP20	pICC1303	FLP recombinase plasmid	38

HeLa cells (ATCC) were maintained in Dulbecco's modified Eagle medium (DMEM) supplemented with 10% fetal bovine serum (FBS) (Sigma-Aldrich) in a 5% CO_2_ incubator at 37°C. Cells were maintained at a maximum density of 1 × 10^6^ cells/ml. The HeLa cells used in this study were authenticated via short tandem repeat profiling in February 2016 (Microsynth).

### Construction of plasmids and strains.

A full list of the oligonucleotides used in this study can be found in Table S1 in the supplemental material.

To permit the use of pWSK29 in ampicillin-resistant *S*. Typhi strains, pWSK29-Spec and pWSK29-Spec-4HA were constructed by amplifying the backbone of pWSK29 (37) and pWSK29-4HA, without the ampicillin resistance cassette, with primers containing NcoI and AvrII restriction sites (primer pair 1 and 2); the *aadA* gene, which confers resistance to spectinomycin, was amplified with primers containing the same restriction sites (primer pair 3 and 4). In addition to digestion with NcoI and AvrII, both products were digested with DpnI prior to ligation.

To create SptP complementation plasmids, SptP from *S*. Typhimurium 14028 (STM14_3477) and *S*. Typhi Ty2 (t2780) and H58 were amplified with primers containing BamHI and KpnI restriction sites (primer pair 5 and 6 and primer pair 7 and 8, respectively) and then cloned into these sites of pWSK29-Spec, generating constructs which constitutively express untagged SptP.

To create HA-tagged SptP, pWSK29-Spec-4HA was amplified with a reverse primer containing the PacI digestion site (primer pair 9 and 10). SptP was again amplified from both *S*. Typhimurium and *S*. Typhi with primers containing BamHI and PacI restriction sites (primer pair 11 and 13 and primer pair 12 and 13, respectively). Both products were digested, and SptP was cloned into the existing BamHI site and the introduced PacI site of pWSK29-Spec-4HA, resulting in constitutively expressed C-terminally tagged SptP-4HA. SptP-4HA expressed from its endogenous promoter was constructed in the same way, with the exception that SptP was amplified from both *S*. Typhimurium and *S*. Typhi with an additional 700 bp upstream from the annotated start codon (primer pair 14 and 13) and ligated into pWSK29-Spec-4HA as described above.

To create SptP-4HA chimeras in which the chaperone-binding domain was switched with that of the other serovar, the first 417 bp of SptP, encompassing the SicP binding domain (amino acid positions 35 to 139), was amplified from the *S*. Typhimurium (primer pair 15 and 16) and *S*. Typhi (primer pair 17 and 18) chromosomes. The *S*. Typhimurium and *S*. Typhi/pWSK29-SptP-4HA plasmids were then amplified without the SicP binding domain (primer pair 19 and 21 and primer pair 20 and 21, respectively), and the two products (an *S*. Typhimurium/pWSK29-SptP-4HA backbone with *S*. Typhi SicP binding and vice versa) were assembled via Gibson assembly (50°C, 30 min) (New England BioLabs).

To determine protein interactions by a bacterial two-hybrid assay, SptP (primer pair 22 and 24 and primer pair 23 and 24 for *S*. Typhimurium and *S*. Typhi SptP, respectively) and SicP (primer pair 25 and 26 and primer pair 25 and 27 for *S*. Typhimurium and *S*. Typhi SicP, respectively) were amplified from both *S*. Typhimurium 14028 and *S*. Typhi Ty2 and cloned into the BamHI and KpnI sites of pKT25 and pUT18C, generating SptP and SicP fused to the C terminus of the T25 and T18 adenylate cyclase fragments.

Gene deletions were constructed via bacteriophage λ red recombination as previously described (38). Primers 28 and 30 were used to amplify the kanamycin resistance cassette of pKD4 with regions of homology to *S*. Typhimurium *sptP*, and primer pair 29 and 30 was used to amplify the kanamycin resistance cassette of pKD4 with regions of homology to S. Typhi *sptP*; primers 30 and 31 were used to create a kanamycin resistance cassette with *sopE* flanking regions; primer pair 32 and 33 was used to create the *sopE2* kanamycin resistance cassette product; and primers 41 and 42 were used to create the kanamycin resistance cassette with regions of *invA* homology. Correct deletion of the gene via insertion of the kanamycin resistance cassette was screened using primers 31 and 32 (*sptP*), 35 and 36 (*sopE*), 39 and 40 (*sopE2*), and 43 and 44 (*invA*). To create the SL1344 Δ*sopE* Δ*sopE2* double mutant strain, the FLP recognition target (FRT)-flanked kanamycin resistance cassette within *sopE* was excised via FLP-mediated recombination (pCP20), generating SL1344 *sopE*::FRT, prior to transformation with the *sopE2* pKD4 PCR product.

### HeLa cell invasion assays.

The invasiveness of the Δ*sptP* and Δ*sopE* strains relative to that of WT Salmonella was determined by gentamicin protection assays. At 24 h prior to infection, HeLa cells were seeded in a 24-well plate at a density of 7 × 10^4^ cells/well. To induce expression of the SPI-1 T3SS, Salmonella strains were either cultured overnight at 37°C and 200 rpm before they were subcultured 1:33 in LB until late exponential phase (optical density at 600 nm [OD_600_], ∼1.8) or cultured overnight at 37°C without shaking. Salmonella was added to the cells at a multiplicity of infection (MOI) of 100:1, and the cells and bacteria were incubated for 15 min for *S*. Typhimurium infections or for 30 min for infections involving both *S*. Typhimurium and *S*. Typhi. The cells were then washed three times with phosphate-buffered saline (PBS), before the addition of DMEM supplemented with 100 μg/ml gentamicin. The cells were further incubated for 1 h and washed with PBS, and the gentamicin concentration was reduced to 20 μg/ml. At the required time points, the cells were again washed three times in PBS before the addition of 0.1% Triton X-100. The cells were incubated for 5 min at room temperature (RT), before serial dilution and plating onto LB agar plates to enumerate the intracellular bacteria.

### Immunofluorescence staining and microscopy.

To compare the ability of the strains to recover the host cytoskeleton postinfection, cells were seeded onto glass coverslips and infected as outlined above. At the required time point, the cells were washed three times in PBS, before fixation with 4% paraformaldehyde for 15 min at room temperature, and were then washed a further three times in PBS. The cells were then quenched in 50 mM ammonium chloride for 10 min at RT, prior to permeabilization in 0.2% Triton X-100 for 4 min. Coverslips were blocked in 0.2% bovine serum albumin (BSA) for 5 min before addition of the primary antibody diluted in 0.2% BSA for 1 h. Intracellular Salmonella bacteria were detected using the CSA-1 antibody (1:200; Insight Biotechnology). The cells were again washed and blocked, prior to addition of the appropriate secondary antibody (1:200 donkey anti-goat immunoglobulin–Cy2; Jackson ImmunoResearch), Hoechst (1:1,000; Sigma), and phalloidin-tetramethyl rhodamine isocyanate (1:100; Invitrogen) for 30 min at room temperature. The coverslips were washed in PBS before they were mounted with ProLong Gold antifade reagent (Invitrogen) and visualized using a Zeiss Axio Observer Z1 microscope at ×100 magnification.

### SPI-1 secretion assays and Western blotting.

To determine the secretion of SptP, Salmonella strains were grown aerobically to late exponential phase in 50 ml LB. The OD_600_ was recorded, and 1 ml bacteria was pelleted and resuspended in 2× SDS loading buffer (1 M Tris, pH 6.8, 2% SDS, 20% glycerol, 5% β-mercaptoethanol, bromophenol blue) in proportion to the OD_600_ reached to normalize bacterial numbers (10 μl of loading buffer per 0.1 OD_600_ unit). The remaining culture was centrifuged for 20 min at 3,300 × *g* before syringe filtering (pore size, 0.2 μm). Five milliliters of the cleared supernatant was precipitated overnight at 4°C following the addition of 10% trichloroacetic acid (TCA). Precipitated proteins were recovered by centrifugation at 20,000 × *g*, followed by two acetone washes. The pellets were left to air dry before the addition of 2× SDS loading buffer in the same volume used for the pellet fractions. To determine SptP stability or intracellular protein levels, the strains were grown aerobically to late exponential phase in 5 ml LB, before the OD_600_ was recorded and 1 ml was pelleted and resuspended in 2× SDS loading buffer. Samples were then heated at 95°C for 10 min. Proteins were separated on 12% acrylamide SDS-polyacrylamide gels, followed by semidry or wet transfer onto a polyvinylidene difluoride membrane (GE Healthcare). The membranes were blocked in 5% milk in PBS–0.05% Tween 20 and blotted with either anti-DnaK 8E2/2 (1:10,000; Enzo Life Sciences), anti-HA (1:1,000; Sigma), or anti-SipD (1:5,000) primary antibodies, followed by the addition of horseradish peroxidase-conjugated secondary antibody (1:10,000; Jackson ImmunoResearch). Following the addition of the EZ-ECL reagent (Geneflow), chemiluminescence was detected using a LAS-3000 imager (Fuji).

### Bacterial two-hybrid and β-galactosidase assays.

E. coli DHM1 was cotransformed with pKT25 and pUT18C constructs in various combinations, and transformants were selected on LB agar plates supplemented with kanamycin and ampicillin. Three colonies from each transformation were subsequently grown overnight with antibiotic selection at 37°C. Ten microliters of these cultures was spotted onto LB agar plates supplemented with kanamycin, ampicillin, 1 mM isopropyl-β-d-thiogalactopyranoside (IPTG), and 0.1 mg/ml of 5-bromo-4-chloro-3-indolyl-β-d-galactopyranoside (X-Gal) and incubated at 30°C for 24 h. Colonies with reconstituted adenylate cyclase activity mediated by interaction of the fused proteins were distinguished by their ability to metabolize X-Gal and subsequent blue coloration (33).

For quantitative analysis, colonies were resuspended in 1.2 ml LB. The OD_600_ was recorded, and the bacteria were pelleted and resuspended in Z buffer (0.06 M Na_2_HPO_4_, 0.04 M NaH_2_PO_4_, 0.01 M KCl, 0.001 M MgSO_4_, 0.05 M β-mercaptoethanol, pH 7). β-Galactosidase assays were then performed as previously described (39).

### Statistical analysis.

Statistical tests were performed using GraphPad Prism (version 7.00) software for Windows (GraphPad Software, San Diego, CA, USA). All data are expressed as the mean ± standard deviation (SD). Significance (*P* < 0.05) was determined either by unpaired *t* test or by one-way analysis of variance (ANOVA) followed by Tukey's *post hoc* test.

## Supplementary Material

Supplemental material
