# Supplementary material for: The Type III Secretion System Effector SptP of Salmonella enterica Serovar Typhi
Source: J Bacteriol. 2017 Jan 30;199(4):e00647-16. doi: 10.1128/JB.00647-16 (PMC5287405; doi:10.1128/JB.00647-16)
Supplement: Supplemental material [file supp_199_4_e00647-16__index.html]

The Type III Secretion System Effector SptP of Salmonella enterica Serovar Typhi — Supplemental material 

# The Type III Secretion System Effector SptP of Salmonella enterica Serovar Typhi

## Supplemental material

- Supplemental file 1 -

  Table S1 (Oligonucleotides) and Fig. S1 (SPI-1 T3SS and invasion)

  PDF, 269K
