# Supplementary material for: The Type III Secretion System Effector SptP of Salmonella enterica Serovar Typhi
Source: J Bacteriol. 2017 Jan 30;199(4):e00647-16. doi: 10.1128/JB.00647-16 (PMC5287405; doi:10.1128/JB.00647-16)
Supplement: Supplemental material [file JB.00647-16_zjb999094317s1.pdf]

Supplementary Table 1. Oligonucleotides used in this study.

	Name	Sequence (Restriction site underlined)
1	pWSK FOR NcoI	catgCCATGGCTGTCAGACCAAGTTTACTC
2	pWSK REV AvrII	catgCCTAGGACTCTTCCTTTTTCAATATTATTG
3	SpecR FOR AvrII	catgCCTAGGATGCGCTCACGCAACTGG
4	SpecR REV NcoI	catgCCATGGCAGGCGTTTAAGGGCACC
5	pWSK sptP Tm FOR BamHI	catgGGATCCATGCTAAGGTATGATGAGAGGAAGTTG
6	pWSK sptP Tm REV KpnI	catgGGTACCTCAGCTTGCCGTCGTCATAAGCAAC
7	pWSK sptP Ty FOR BamHI	catgGGATCCATGCTAAAGTATGAGGAGAGAAAATTGAATAATTTAAC
8	pWSK sptP Ty REV KpnI	catgGGTACCTCAGCTTGCCGTCGTCATAAG
9	pWSK29-Spec-Rv	ggatccactagttctagag
10	pWSK29-Spec-StoD-4HA-PacI-Fw	gcTTAATTAAGggattagcgatgaaatatgc
11	pWSK4HA sptP Tm FOR NotI	catgGCGGCCGCgATGCTAAAGTATGAGGAGAGAAAATTGAATAATTTAAC
12	pWSK4HA sptP Ty2 FOR NotI	catgGCGGCCGCgATGCTAAGGTATGATGAGAGGAAGTTG
13	pWSK4HA sptP Ty2 Tm REV PacI	catgTTAATTAAGCTTGCCGTCGTCATAAGC
14	pWSK4HA sptP +700 FOR	catgGCGGCCGCGGTCAGACCCATCTGGTTGAG
15	GIB Tm SicP FOR	gcggtggcggccgcgATGCTAAAGTATGAGGAGAG
16	GIB Tm SicP REV	gcattcatgacaccaacATTATCCTTGCTCTTAATAAGGTTTATAAAC
17	GIB Ty2 SicP FOR	gcggtggcggccgcgATGCTAAGGTATGATGAGAG
18	GIB Ty2 SicP REV	cattcctgacaccaacATTATCCTTGTTCTTAATAAGATTTATAAAC
19	GIB Tm pWSK FOR	GTTGGTGTCAGGAATGCCGCTTTAGTCATAAAAGGCGGC
20	GIB Ty2 pWSK FOR	GTTGGTGTCATGAATGCCGCTTTAGTCATAAAAGGCGGCGATAC
21	GIB Ty2 Tm pWSK REV	CGCGGCCGCCACCGCGGT
22	B2H sptP Tm FOR BamHI	catgGGATCCgTTGAATAATTTAACGTTGTCTTCGTTTTC
23	B2H sptP Ty2 FOR BamHI	catgGGATCCgTTGAATAATTTAACATTGTCTTCATTTTC
24	B2H sptP Tm Ty2 REV KpnI	catgGGTACCTCAGCTTGCCGTCGTCATAAG
25	B2H sicP Tm Ty2 FOR BamHI	catgGGATCCgTTGCAAGCACACCAGGATATTATC
26	B2H sicP Tm REV KpnI	catgGGTACCTCATACTTTAGCATATTCCTGCAGTATG

27	B2H sicP Ty2 REV KpnI	catgGGTACCTCATACCTTAGCATATTCCTGTAGTATG
28	ΔsptP Tm FOR	GAATAATTTAACGTTGTCTTCGTTTTCAAAAGTTGGTGTGTCGAATGATGCCCGAgtgtaggctggagctgcttc
29	ΔsptP Ty2 FOR	CATTGTCTTCATTTTCAAAATCTGGCGTGTCGAGTGATACCCGGCTATATATTGCgtgtaggctggagctgcttc
30	ΔsptP Tm Ty2 REV	CATAAGCAACTGGGCTTGCATTGCTTTTAGTTGTACAAACTGTGAGgcgtcttccCATATGAATATCCTCCTTAG
31	SptP UP	CATGCAATTACCGATCTGAC
32	SptP DOWN	CAGAAATAGCTTACTTTCAGATAGTTC
33	ΔsopE Tm Ty2 FOR	CTTTATCTCCCCAGAATTTTAGAATCCAAAAACAGGAAACCACACTACgtgtaggctggagctgcttc
34	ΔsopE Tm Ty2 REV	GTATATATTTATTAGCAATGTTTTCTAGTATCAGTTGGAATTGCTGTGCATATGAATATCCTCCTTAG
35	SopE UP	GCTCAACGATCAGCTCACACTC
36	SopE DOWN	GGATACAGCTAACCCAGACTG
37	ΔsopE2 Tm FOR	CACTATCCACCCAGCACTACAGAATCCATAGAAGTGACGTTGAACCAGGTGTAGGCTGGAGCTGCTTC
38	ΔsopE2 Tm REV	GATACTTATTCGCAATATTTTCTATAGTTAACTGAAATTGTTGTGGCGCATATGAATATCCTCCTTAG
39	SopE2 UP	CCTGTTCAGGATTGTCCCG
40	SopE2 DOWN	GCTGTCTGGTTGTGCAGG
41	ΔinvA Ty2 FOR	ACTTAACAGTGCTCGTTTACGACCTGAATTACTGATTCTGGTACTAATGGGTGTAGGCTGGAGCTGCTTC
42	ΔinvA Ty2 REV	GCTATCTGCTATCTCACCGAAAGATAAAACCTCCAGATCCGGAAAACGACCCATATGAATATCCTCCTTAG
43	InvA UP	GTTACGTAGTATGACCGATATTGC
44	InvA DOWN	CCTCCAATTAATGAAGGATCGC

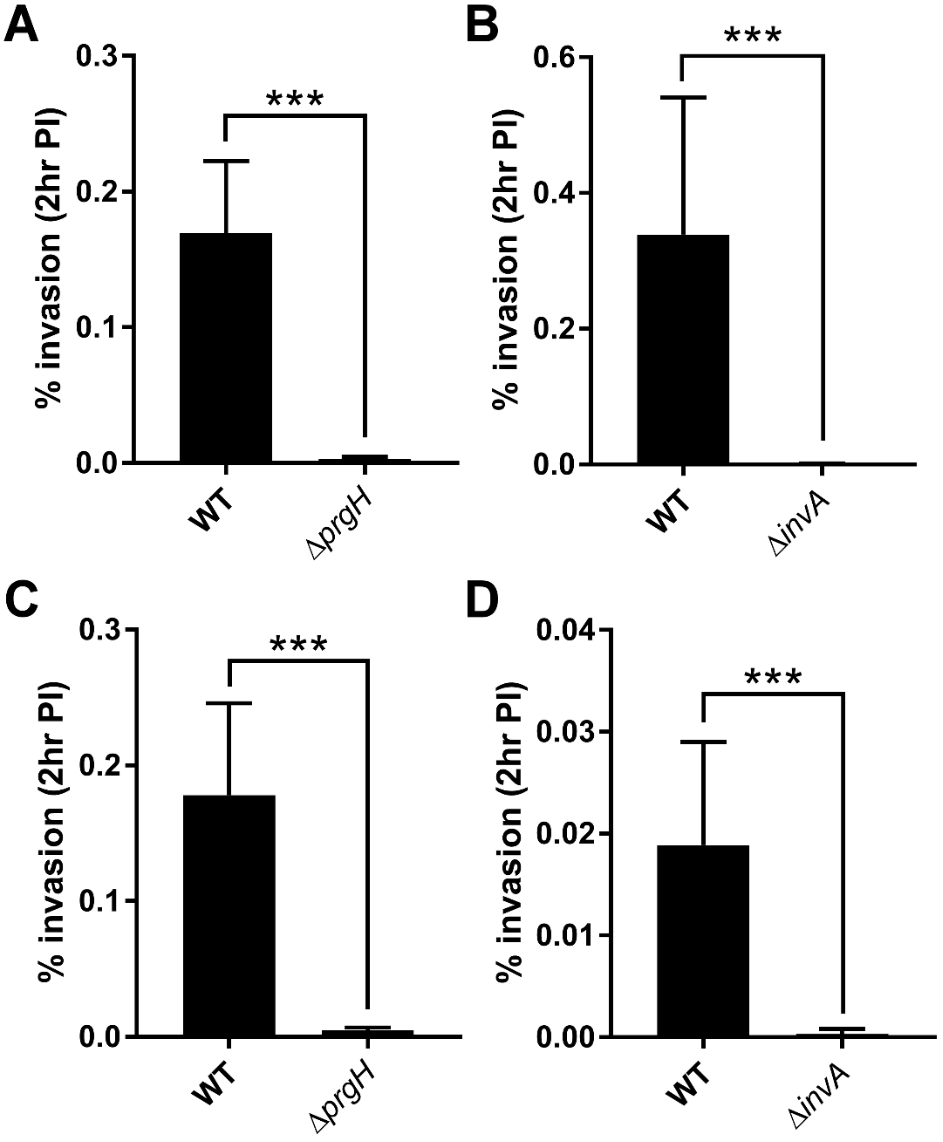

Supplementary Figure 1. The SPI-1 T3SS is required for invasion. WT and SPI-1 deficient *Salmonella* were grown either aerobically to late exponential phase (subculture) (**A** and **B**) or to stationary phase under microaerobic conditions (static overnight) (**C** and **D**) and added to HeLa cells at an MOI 100. For exponentially grown bacteria, cells were infected for 15 min for WT and Δ*prgH* S. Typhimurium (**A**), and 1 h with WT and Δ*invA S*. Typhi (**B**). For stationary phase bacteria, cells were infected for 30 min with both *S*. Typhimurium (**C**) and *S*. Typhi (**D**). The percentage of intracellular bacteria at 2 h post-infection relative to the inoculum added is shown. n=3, error bars show SD. Invasion rates of strains were compared by t-*test* (*** = P < 0.001)
